# Identification of Novel Locus *RsCr6* Related to Clubroot Resistance in Radish (*Raphanus sativus* L.)

**DOI:** 10.3389/fpls.2022.866211

**Published:** 2022-05-19

**Authors:** Caixia Gan, Chenghuan Yan, Wenxing Pang, Lei Cui, Pengyu Fu, Xiaoqing Yu, Zhengming Qiu, Meiyu Zhu, Zhongyun Piao, Xiaohui Deng

**Affiliations:** ^1^Hubei Key Laboratory of Vegetable Germplasm Enhancement and Genetic Improvement, Institute of Economic Crops, Hubei Academy of Agricultural Sciences, Wuhan, China; ^2^College of Horticulture, Shenyang Agricultural University, Shenyang, China; ^3^College of Chemistry and Life Science, Chifeng University, Chifeng, China

**Keywords:** radish, *Plasmodiophora brassicae*, clubroot disease, bulked segregant analysis, RNA-seq

## Abstract

Clubroot is a devastating disease that causes substantial yield loss worldwide. However, the inheritance and molecular mechanisms of clubroot resistance during pathogen infection in radish remain largely unclear. In this study, we investigated the inheritance of clubroot resistance in the F_2_ population derived from crossing clubroot-resistant (CR) and clubroot-susceptible inbred lines “GLX” and “XNQ,” respectively. Genetic analysis revealed that a single dominant gene controlled the clubroot resistance of “GLX” with a Mendelian ratio of resistance and susceptibility of nearly 3:1. Bulked segregant analysis combined with whole-genome resequencing (BSA-seq) was performed to detect the target region of *RsCr6* on chromosome Rs8. Linkage analysis revealed that the *RsCr6* locus was located between two markers, HB321 and HB331, with an interval of approximately 92 kb. Based on the outcomes of transcriptome analysis, in the *RsCr6* locus, the *R120263140* and *R120263070* genes with a possible relation to clubroot resistance were considered candidate genes. In addition, three core breeding materials containing the two reported quantitative trait loci (QTLs) and our novel locus *RsCr6* targeting clubroot resistance were obtained using marker-assisted selection (MAS) technology. This study reveals a novel locus responsible for clubroot resistance in radishes. Further analysis of new genes may reveal the molecular mechanisms underlying the clubroot resistance of plants and provide a theoretical basis for radish resistance breeding.

## Introduction

Clubroot disease, caused by *Plasmodiophora brassicae*, is a severe disease in cruciferous crops worldwide. *P. brassicae* affects most cruciferous crops, such as radish (Kamei et al., 2010; Gan et al., 2019), Chinese cabbage (Piao et al., 2009; Li et al., 2016), cabbage (Piao et al., 2009), pak choi (Chen et al., 2016), and canola (Zhan et al., 2017). Clubroot leads to the formation of swellings or galls on the roots, which can ultimately cause wilting and premature death, leading to an annual crop yield reduction of 10–15% worldwide (Voorrips et al., 1997; Dixon, 2006). It is now widespread in all parts of China, such as Hubei, Hunan, Yunnan, Sichuan, Guangxi, Guangdong, Liaoning, Heilongjiang, Jilin, Shandong, and other provinces (cities and autonomous regions). Notably, clubroot can significantly affect the edible and commercial value of radish, consumed as a taproot vegetable. In 2017, the sowing area of radish in China reached 1.3 million hm^2^. Hubei has the largest planting area of radish, accounting for one-tenth of China's radish production area (Bao et al., 2019). Among these, mountain vegetables in Hubei Province have developed rapidly in recent years (Wang et al., 2013). In 2003, an outbreak of clubroot disease happened in Huoshaoping, Changyang County (Hubei Province, China). It quickly spread, becoming the biggest problem faced by the industrialization of alpine vegetables and farmers' income increase in this area (Gan et al., 2016, 2017), which accounted for a 30–50% reduction in the yield and quality of radish. Therefore, it is necessary to identify an effective clubroot prevention and control strategy for radish plants. Breeding clubroot-resistant (CR) cultivars and practicing crop rotation are the most effective methods for managing clubroot (Pang et al., 2018).

It is important to breed CR cultivars using marker-assisted selection (MAS) technology for preventing clubroot disease. CR genes have been studied and identified in the A-genome of *Brassica rapa*, such as *Crr1* (Suwabe et al., 2003), *Crr2* (Suwabe et al., 2003), *Crr3* (Saito et al., 2006), *Crr4* (Suwabe et al., 2006), *CRc* (Sakamoto et al., 2008), *CRk* (Sakamoto et al., 2008), *CRa* (Ueno et al., 2012), *CRd* (Pang et al., 2018), *PbBa1.1, PbBa3.1, PbBa3.2, PbBa3.3, PbBa8.1* (Chen et al., 2013), *Rcr1* (Chu et al., 2014), *Rcr2* (Huang et al., 2017), *Rcr4, Rcr8*, and *Rcr9* (Yu et al., 2017). Quantitative trait loci (QTLs) involving race-specific resistance were detected in the C genome of *B. oleracea*, such as *CR2a* and *CR2b* (Landry et al., 1992), *pb-3* and *pb-4* (Voorrips et al., 1997), *pb-Bo1* (Rocherieux et al., 2004) *pb-Bo*(Anju)1, *pb-Bo*(Anju)2, *pb-pb-Bo*(Anju)3, *pb-Bo*(Anju)4, and *pb-Bo*(GC)1 (Nagaoka et al., 2010). At least 20 QTLs involved in CR have been identified in the A and C genomes (Chang et al., 2019). Moreover, *Crs1, RsCr1, RsCr2, RsCr3, RsCr4*, and *RsCr5* have been identified in radish (Kamei et al., 2010; Gan et al., 2019). Of these reported CR genes, *CRa* and *Crr1* have been cloned and identified to encode Toll-Interleukin-1 receptor/nucleotide-binding site/leucine-rich-repeat (TIR-NBS-LRR) proteins (Ueno et al., 2012).

The identified CR loci and their molecular markers have greatly accelerated the breeding of CR cultivars in Brassica crops. CR genes have been successfully transformed into Chinese cabbage through MAS (Yoshikawa, 1981; Zhang et al., 2012). However, breeding of CR cultivars using the identified CR locus has not yet been reported in radish. Therefore, identifying more CR genes/loci conferring resistance to different pathotypes of *P. brassicae* from distinct geographical regions is essential for breeding CR cultivars in radish. Pang et al. (2020) reported the pathotype diversity of *P. brassicae* and its distribution in China. HBHSP-91 was collected from Huoshaoping, the most prominent radish farming region in Hubei Province, and was identified as pathotype 4 and Pb10 according to Williams” clubroot differential set and the SCD system, respectively (Pang et al., 2020).

In this study, CR and susceptible inbred lines, “GLX” and “XNQ,” respectively, of radish were used to develop a new segregation population and reveal the genetic basis underlying clubroot resistance. We aimed to identify the clubroot resistance locus and develop critical molecular markers for breeding CR cultivars by predicting and analyzing candidate genes in the target region. Our study sheds light on the molecular mechanisms dominating clubroot resistance in radish and provides user-friendly molecular markers for breeding CR cultivars in the future.

## Materials and Methods

### Plant Materials and Inoculation

Changyang has a large radish planting area in China, with the most severe radish clubroot disease. A preliminary study identified the *P. brassicae* (HBHSP-91) collected in Huoshaoping, Changyang County as pathotype Pb10 (Pang et al., 2020). In this study, inoculation was performed based on an established infection method (Gan et al., 2019). A disease resistance investigation of radish germplasm resources was performed in both greenhouse and field planting. Disease resistance was determined by a single spore of Pb10 (Pang et al., 2020) inoculation in a greenhouse at the Wuhan Institute of Economic Crops, Hubei Academy of Agricultural Sciences. Field testing was conducted in the infected area of Huoshaoping in Changyang County, Hubei Province (Gan et al., 2017) during the production season when temperatures ranged from 18 to 28°C. The susceptible XNQ (disease index [DI] = 87.5) and resistant GLX (DI = 0) lines, both advanced-generation inbred radish lines, were obtained. The susceptible parent “XNQ” was crossed with the resistant parent “GLX” to obtain F_1_. The resultant F_1_ was self-crossed to develop an F_2_ segregating population with 823 individuals to identify the CR locus. In this study, all materials were grown in a greenhouse at the Hubei Academy of Agricultural Sciences (Wuhan, China).

### Inoculation and Phenotype Identification

Phenotype identification of parents, F_1_ and F_2_, was carried out in the greenhouse of the Hubei Academy of Agricultural Sciences (Wuhan, China). Plants were grown in 50-well-multipots. The plants were inoculated with the *P. brassicae* pathogen at the stage of two cotyledons and one euphylla leaf and cultured at 25°C under a 16 h light/8 h dark photoperiod. Phenotype identification of the parents and F_1_ was repeated three times, each time with more than 15 seedlings. More than 1,000 F_2_ plants were used in the CR tests. Disease symptoms were used to calculate the DI on a scale of 0–4 (Gan et al., 2019). The disease symptoms in the roots of each plant were evaluated as follows: grade 0, no clubs; grade 1, a few small or separate clubs on the lateral roots or the main root; grade 2, intermediate symptoms; grade 3, numerous clubs on the main roots; and grade 4, clubs rotten or plants died. Investigations were performed at 6 weeks after pathogen inoculation, and all the plants with galls (slight, moderate, or large) on the main or lateral roots were considered infected.

### BSA-Seq Analysis

Bulked segregant analysis (BSA) and whole-genome resequencing, named BSA-seq, and were employed to detect the clubroot resistance locus in radish. Two pools were constructed using equal amounts of leaf tissues from the F_2_ individuals, with 50 susceptible individuals constituting the S-pool and 50 resistant individuals comprising the R-pool. The genomic DNA of the two pools was isolated using a previously reported cetyl trimethylammonium bromide (CTAB) method (Webb and Knapp, 1990).

Whole-genome resequencing of the two pools was performed on the Illumina HiSeq platform using an approximate 400 bp library (Genoseq, Wuhan). Approximately 15 Gb of clean data were obtained from the S and R-pools through sequencing (Genome Sequence Archive, GSA: PRJCA008465). Additionally, the two parental lines were subjected to resequencing with 30 × depth. As previously described (Yan et al., 2020), BSA-seq analysis was performed using a user-friendly script. The clean reads from two pools were aligned against the long-read reference genome “QZ-16” (ENA number: PRJEB37015) by Bowtie (Langmead and Salzberg, 2012). Single nucleotide polymorphism (SNP) calling was conducted using the SAMtools software (Li et al., 2009). Low-quality SNPs were automatically removed using the software. The SNP index was calculated using the method reported by Takagi et al. (2013). The critical parameter Δ(SNP-index) was calculated by subtracting the SNP index of the R-pool from that of the S-pool. The SNP index was plotted using a 50 kb window size and a 10 kb window step. Then, 95% confidence intervals (*CI*s) were calculated to identify the significant resistance gene locus.

### Molecular Marker Development

Based on the BSA analysis, we developed cleaved amplified polymorphic sequence (CAPS) markers for further mapping. The development pipeline for the CAPS markers followed our previously reported method (Yan et al., 2019). Based on two major QTLs on chromosomes Rs5 and Rs8, we developed InDel markers, according to the method reported in our previous study (Yan et al., 2022). Primers for these molecular markers were designed using Primer3Plus (https://primer3plus.com/) (Table S5). PCR amplification was conducted in a 20 μl volume containing 10 μl 2xES Taq Mixes (CWBIO, Taizhou, China), 1 μl forward primer, 1 μl reverse primer, 1 μl template, and 7 μl ddH_2_O. PCR reactions followed the process: 94°C for 4 min, followed by 40 cycles at 94°C for 15 s, 53°C for 20 s, 72°C for 45 s, and a final extension step at 72°C for 5 min. These PCR products were digested for 8 h in a 10 μl reaction volume containing 5 μl PCR products, 1 μl 10 × CutOne buffer, 0.3 μl of the enzyme, and 3.7 μl of ddH_2_O.

### Genetic Mapping Analysis

To rapidly identify the clubroot resistance locus, we used the BSA-seq to map the target region of *RsCr6*. We randomly selected 116 progenies from the F_2_ population of “GLX” crossed with “XNQ” in the target region. Two flanking markers delimiting the *RsCr6* locus were used to screen all F_2_ progenies. To fine-map the *RsCr6* gene, additional molecular markers were developed between the two flanking markers. These markers were subsequently used to identify recombinants from the F_2_ population.

### Transcriptome Analysis

A comparative transcriptome analysis (GSA, PRJCA008464) of two parental lines “XNQ” and “GLX,” was performed to identify the differentially expressed genes (DEGs) in resistance plants vs. susceptible plants. The taproots of “XNQ” and “GLX” were sampled at 0 and 3 dpi. The tissues from six individuals were mixed into one sample after inoculation with the pb10 pathotype, and each treatment had three replicates. Total RNA was extracted from these samples using the Trizol method (Life Technologies, Carlsbad, USA). RNA-seq was performed using an Illumina HiSeq platform (Genoseq, Wuhan, China). The quality of the transcriptome data was assessed using principal component analysis (PCA). Expression levels were represented by the value of transcripts per kilobase per million mapped reads (TPM). DEGs were identified using the R package DEseq2 (Love et al., 2014) with a threshold of |log2FoldChange| > 1 and adjusted *p* <0.01. Gene Ontology (GO) and Kyoto Encyclopedia of Genes and Genomes (KEGG) enrichment analyses were performed to identify the biological functions and metabolic pathways of the DEGs. A heatmap of DEGs was generated using the TBtools software (Chen et al., 2020).

## Results

### Phenotype Evaluation and Genetic Analysis

**Figure 1:**
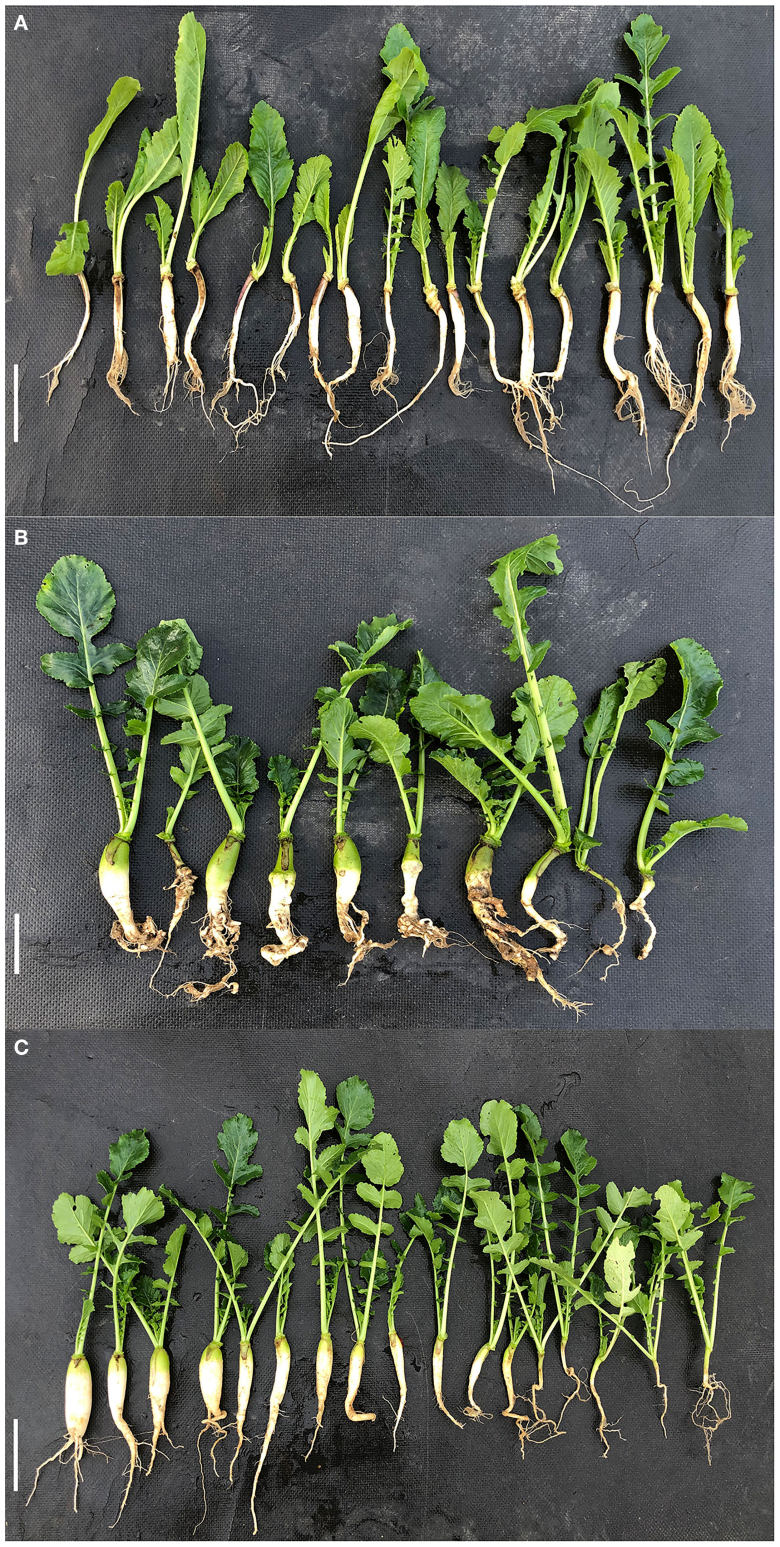
Phenotype identification of two parental radish lines and their crossed offspring after inoculation with Pb10. **(A)** Resistance parent “GLX” (P1). **(B)** Susceptible parent “XNQ” (P2). **(C)** Phenotypes of F_1_ offspring individuals. Bar scale = 10 cm.

Two representative radish cultivars, the resistant “GLX” (Figure 1A) and the susceptible “XNQ” (Figure 1B), were utilized to analyze the inheritance of clubroot resistance in radish. These two cultivars were crossed to obtain F_1_, self-crossing of which gave rise to the F_2_ population. A total of 27 F_1_ offspring exhibited resistance to clubroot in radish (Figure 1C). Among 823 F_2_ progenies, 637 individuals were resistant and 186 individuals were susceptible fitting to a segregation ratio of 3:1 (χ^2^ = 2.933, *p* = 0.0868 > 0.05, Table 1), which indicated that clubroot resistance was controlled by a single dominant locus in the newly segregated population.

**Table 1:** Genetic analysis of P_1_, P_2_, F_1_, and F_2_ populations inoculated with Pb10 pathotype.

**Materials**	**Resistant plants**	**Susceptible plants**	χ^2*^	P^*^
P_1_	28	0		
P_2_	0	29		
F_1_	27	0		
F2	637	186	2.933	0.0868

Notes: “^*^” indicates χ^2^ = 3.841, p = 0.05.

### Prediction of Candidate Region Controlling Clubroot Resistance by BSA-Seq

In the present study, the candidate region controlling clubroot resistance was screened using BSA-seq. Two pools, S and R, were constructed with 50 susceptible and 50 resistant individuals, respectively. Furthermore, 17,582,276,637 and 18,295,677,463 bp clean reads were filtered from the raw data obtained from the S-pool and R-pool, respectively, *via* whole-genome resequencing (Table S1). The overall alignment rates of the clean reads from the S-pool and R-pool against the reference genome were 85.88 and 87.50%, respectively. A total of 6,000,723 differential SNPs were identified between the S- and R-pools. Based on a threshold of Δ(SNP-index) > 0.4, we further filtered the SNPs and obtained 171,987 high-quality SNPs.

**Figure 2:**
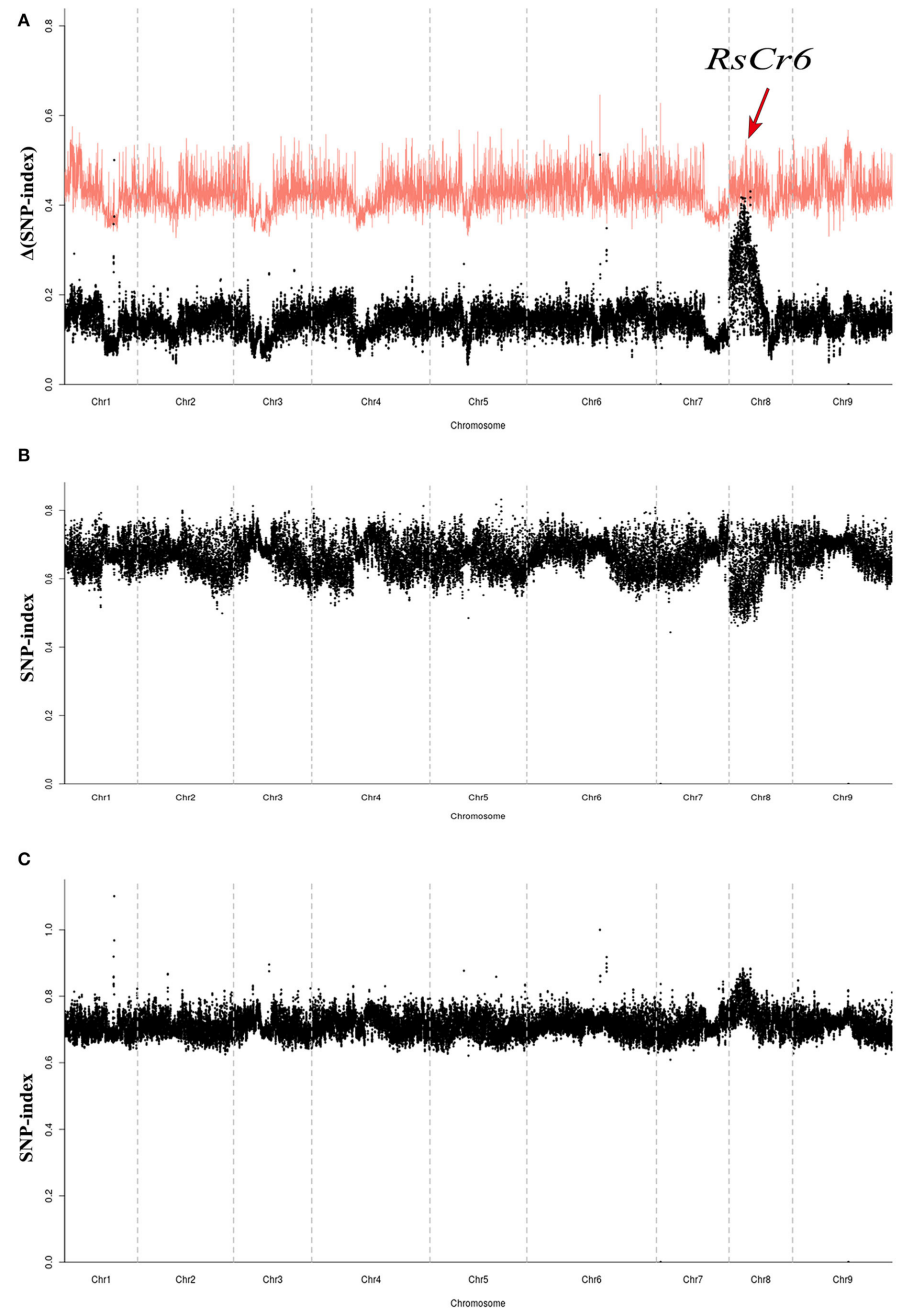
Identification of novel quantitative trait locus (QTL) related to clubroot resistance in radish. **(A)** Plot of Δ(SNP-index) value based on S-pool and R-pool data against the reference genome “QZ-16.” The target peak (red arrow) of the novel clubroot resistance locus *RsCr6* was identified on chromosome Rs8. The confidence interval (*CI*) is indicated in red (*p* <0.05). **(B)** Distribution of single nucleotide polymorphism (SNP)-index value based on S-pool data. **(C)** Distribution of SNP-index value based on R-pool data.

The Δ(SNP-index) value was calculated based on the SNP indices from S- and R-pools and analyzed against the long-read radish genome “QZ-16.” The Δ(SNP index) value distribution was investigated in the genome (Figure 2). A single peak was identified on chromosome Rs8 (5,000,001–8,500,000 bp) with statistical significance (*p* <0.05) (Figure 2A) and was considered as a candidate region associated with clubroot resistance, which was designated as *RsCr6*.

### Genetic Mapping of *RsCr6* Gene

**Figure 3:**
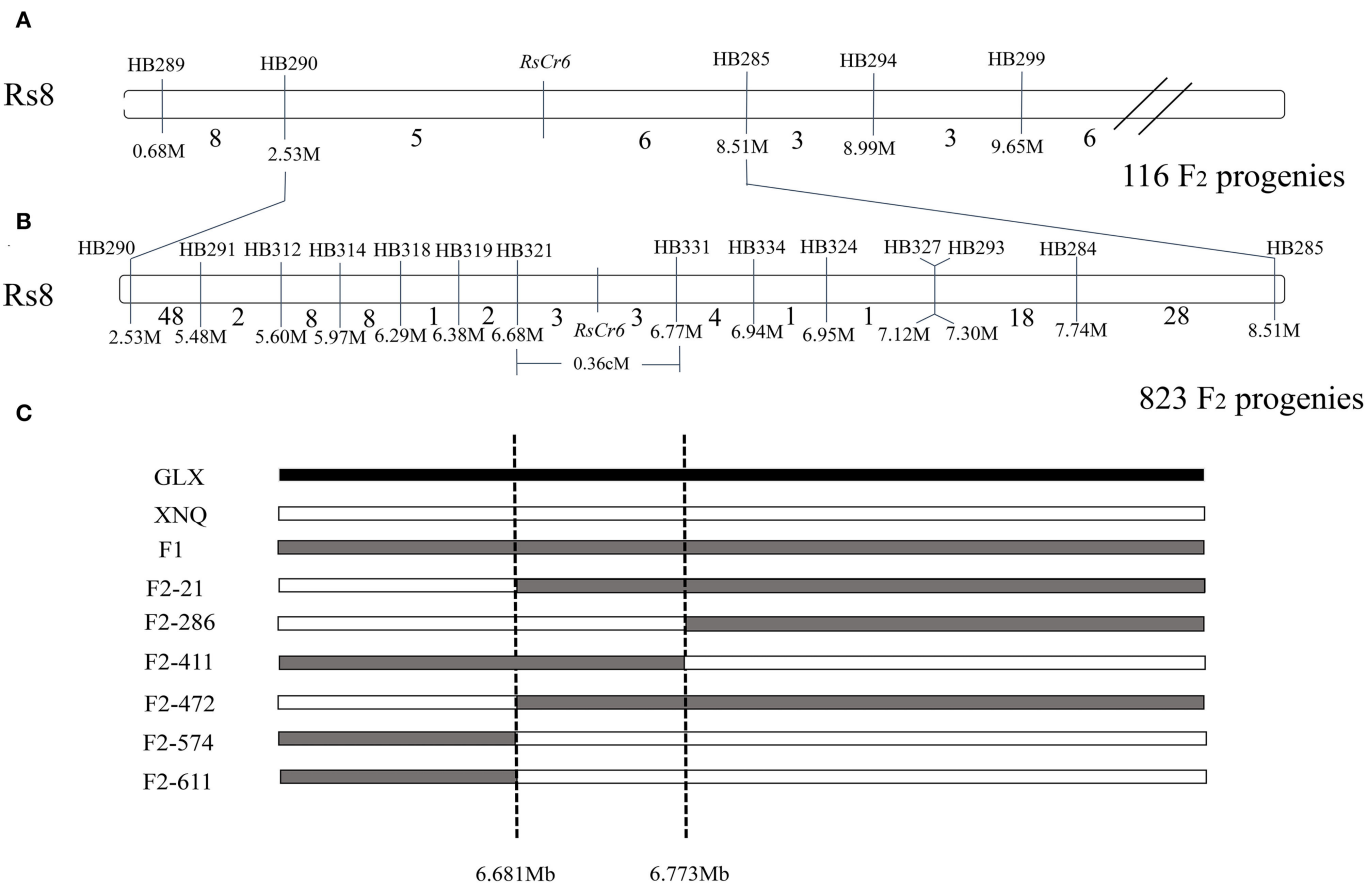
Mapping of clubroot resistance gene *RsCr6*. **(A)** Preliminary mapping of *RsCr6* between molecular markers HB290 and HB285 using 116 F_2_ individuals. **(B)** Fine mapping of *RsCr6* between molecular markers HB321 and HB331 using 823 F_2_ individuals. The numbers between adjacent markers represent the number of recombinants. The numbers below marker names represent the physical position for corresponding markers. **(C)** Diagram of recombinants in a narrow interval between markers HB321 and HB331. “M” and “Mb” represent million bases; and genetic distance is showed in cM.

In the present study, traditional genetic mapping was performed to validate the *RsCr6* locus. Five CAPS markers, such as HB289, HB290, HB285, HB294, and HB299 around the *RsCr6* locus, were developed using our previously reported method (Yan et al., 2020). Furthermore, five CAPS makers were subjected to preliminary mapping, and a total of eleven recombinants were obtained from 116 randomly selected progenies from the F_2_ population. Thus, the *RsCr6* gene was located between the molecular markers HB290 and HB285 (Figure 3A). To narrow the target region further, we used two flanking markers, HB290 and HB285, to screen 823 F_2_ progenies. A total of 72 recombinants between the HB290 and *RsCr6* loci and 55 recombinants between the HB285 and *RsCr6* loci were identified. We further designed 12 CAPS markers to fine-map the *RsCr6* locus. Finally, the *RsCr6* locus was fine-mapped between two molecular markers: HB321 (6,681,758 bp) and HB331 (6,773,184 bp) (Figure 3B). Two flanking markers, HB321 and HB331, shared three recombinants, respectively, with the *RsCr6* gene (Figure 3C). Based on the physical positions of the two markers, *RsCr6* was delimited in a narrow interval of approximately 92 kb on chromosome Rs8 (Figure 3C), with a genetic distance of 0.36 cM.

### Comparative Transcriptome Analysis of Resistance and Susceptible Lines

**Figure 4:**
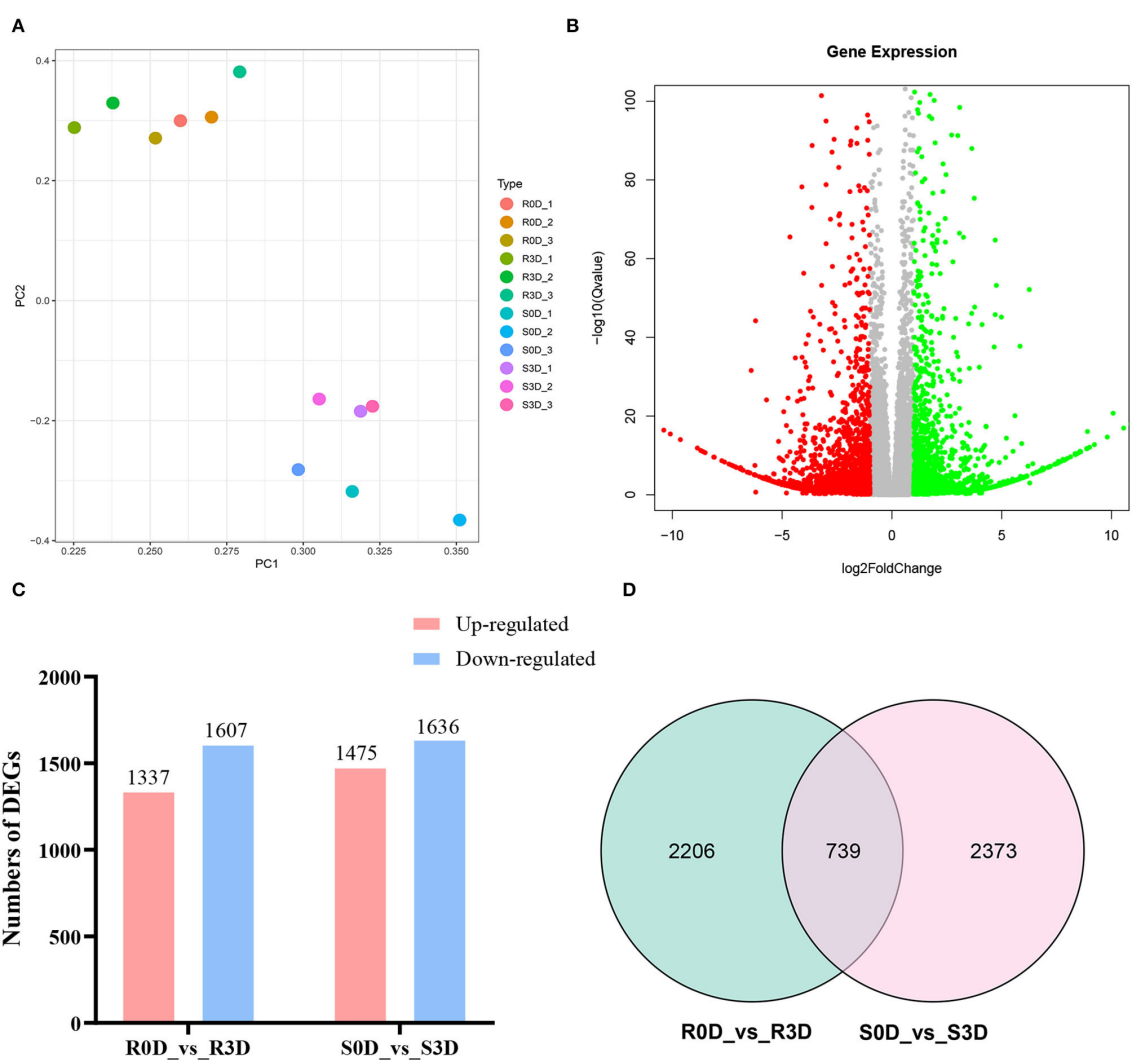
Comparative transcriptome analyses for differentially expressed genes (DEGs) in the two parent lines after inoculation with Pb10. **(A)** Principal component analysis (PCA) of 12 samples. **(B)** Volcano plot of DEGs in resistant parent group of R0D vs. R3D. **(C)** Numbers of DEGs in the two groups of R0D vs. R3D and S0D vs. S3D. **(D)** The Venn diagram of DEGs in the two groups of R0D vs. R3D and S0D vs. S3D. “R” represents the resistant parent lines; “S” represents the susceptible parent lines; “0D” indicates the uninoculated samples; and “3D” indicates that the samples have been collected at 3 days after inoculation with the Pb10 pathotype.

From 0 to 3 days after inoculation, *P. brassicae* could complete the root hair and cortex infections in susceptible materials, while only the root hair could be infected in the resistant materials (Liu et al., 2020). Therefore, we considered that the resistance genes played a key role from day 0 to day 3 of inoculation. A comparative transcriptome sequencing was performed between resistance parent “GLX” and susceptible parent “XNQ” at 0 and 3 days post-inoculation (dpi) to identify DEGs. First, the PCA of the 12 samples was conducted, which revealed that each sample exhibited good reproducibility (Figure 4A). Subsequently, the DEGs were identified and analyzed using DEseq2 (Love et al., 2014). A total of 3,111 DEGs, including 1,636 downregulated and 1,475 upregulated, were identified in the comparison of susceptible radish “XNQ” at 0 dpi vs. at 3 dpi (S0D_vs._S3D), and 2,944 DEGs including 1,607 downregulated and 1,337 upregulated were detected in the comparison of resistance radish “GLX” at 0 dpi vs. 3 dpi (R0D_vs._R3D, Figures 4B,C). The Venn diagram shows the distribution of DEGs in the two parental lines (Figure 4D). A total of 739 DEGs were shared between two pairwise treatment comparisons. We detected 2,206 DEGs unique to the comparison of R0D_vs._R3D, which excluded 739 DEGs at the intersection of two pairwise treatment comparisons. Based on the differences in the genetic backgrounds of the two parents, we considered that these 2,206 unique DEGs in R0D_vs._R3D might be involved in the response to clubroot resistance.

### Candidate Gene Identification of *RsCr6* Based on Comparative Transcriptome Analysis

**Figure 5:**
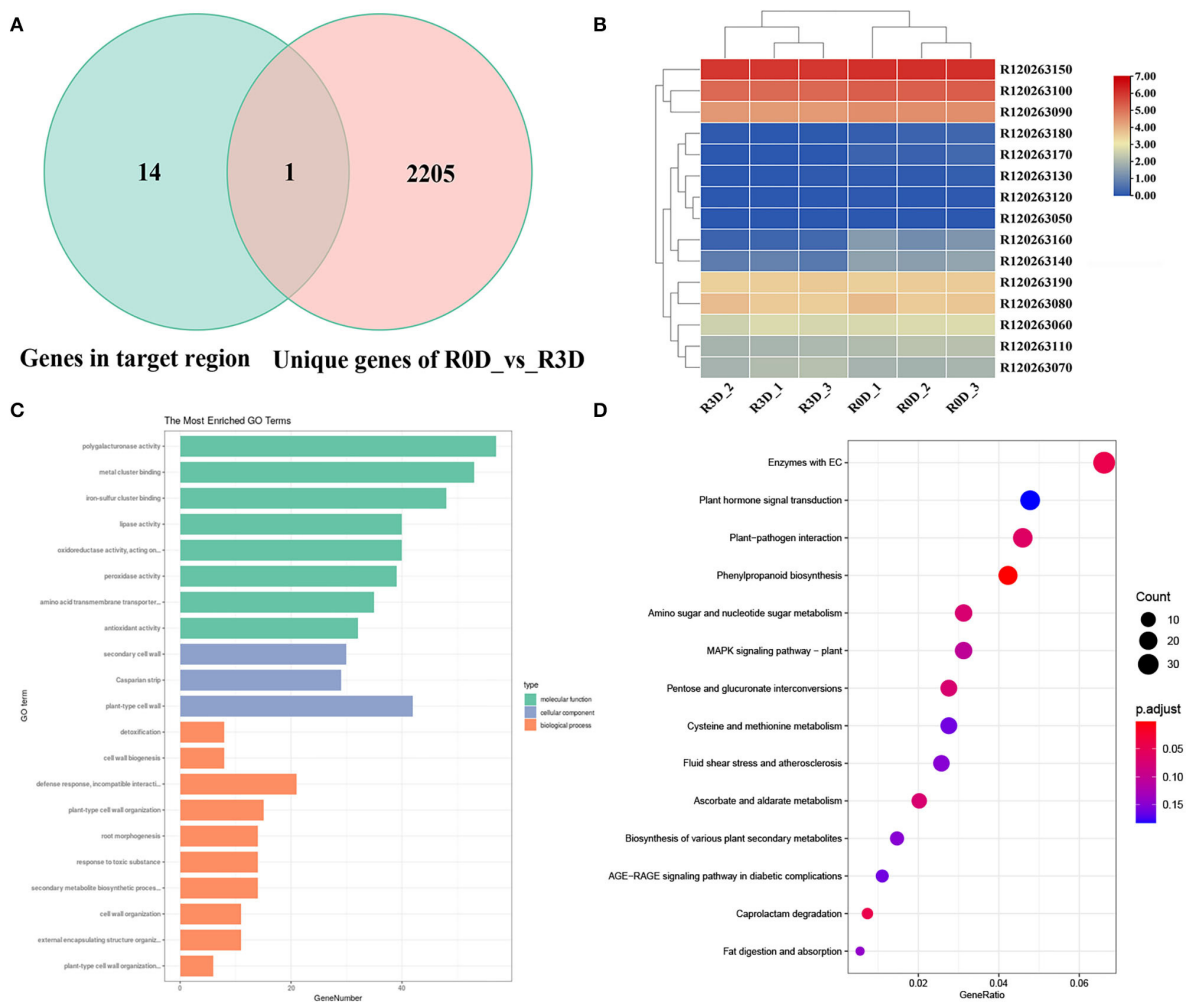
Gene Ontology (GO) enrichment and Kyoto Encyclopedia of Genes and Genomes (KEGG) pathway analyses of DEGs in clubroot resistant radish. **(A)** The Venn diagram of 2,206 unique DEGs in the comparison between R0D and R3D and genes in the *RsCr6* locus. **(B)** Heatmap of a target region in the *RsCr6* locus. Expression levels were calculated using log2-scaled TPM values. TPM, transcripts per kilobase of exon model per million mapped reads. **(C)** GO enrichment analysis of 2,206 unique DEGs in the comparison between R0D and R3D. **(D)** KEGG pathway analysis of 2,206 unique DEGs in the comparison between R0D and R3D.

The candidate gene of *RsCr6* was screened based on comparative transcriptome data and predicted genes in the target region. In the delimited 92 kb region of the *RsCr6* locus, we discovered 15 predicted genes between the molecular markers HB321 and HB331 against the “QZ-16” annotation file (Table 2). Of the 15 predicted genes, only one, *R120263140*, was differentially expressed in the comparison of R0D_vs._R3D (Figure 5A). Heatmap results showed that *R120263140* was significantly downregulated at 3 dpi in the resistance radish “GLX,” compared with 0 dpi (Figure 5B). *R120263140* likely encodes a MIZU-KUSSEI-like protein that shares 88.1% amino acid identity with AT4G39610. We found that another gene, *R120263160* belonging to CYP96A, was also downregulated at 3 dpi in the resistance radish “GLX.” *R120263160* was involved in response to light stimuli. In addition, *R120263160* was identified in the 739 DEG intersections between R0D_vs._R3D and S0D_vs._S3D. In the target region, *R120263070* encodes a probable disease resistance protein named RPP1, which might play a role in resistance processes. However, no differential expressions were observed between the two pairwise comparisons. Together, our analyses revealed that *R120263140* and *R120263070* were likely candidate genes related to *RsCr6*.

**Table 2:** Characteristics of 15 predicted genes between molecular markers HB321 and HB331.

**Gene name**	**Start**	**Stop**	**Length (bp)**	**Identifier**
R120263190	6680212	6684594	2,349	S-adenosylmethionine carrier 1
R120263180	6685901	6687286	1,386	Alkane hydroxylase MAH1
R120263170	6692161	6693642	1,482	Alkane hydroxylase MAH1
R120263160	6694498	6695943	1,446	Alkane hydroxylase MAH1
R120263150	6697610	6701615	3,669	Coatomer subunit alpha-1
R120263140	6704335	6705105	771	Protein MIZU-KUSSEI 1
R120263130	6706107	6707153	1,047	F-box protein
R120263120	6711080	6713952	1,746	Glutathione hydrolase 2
R120263110	6715437	6718546	1,836	Alanine–glyoxylate aminotransferase 2 homolog 1
R120263100	6723427	6725506	1,812	Apoptotic chromatin condensation
R120263090	6726232	6730510	1,947	Unknown protein
R120263080	6735515	6736399	885	Unknown protein
R120263070	6748863	6749951	549	Probable disease resistance protein, RPP1
R120263060	6754044	6757745	1,488	Protein SCO1 homolog 2
R120263050	6762254	6766077	1,743	F-box/kelch-repeat protein

### Pathways Activated by Clubroot Pathogen Infection in Radish

To identify the functions of DEGs, GO enrichment analysis of the unique 2,206 DEGs in the comparison R0D_vs._R3D was performed against the GO database with *p* ≤ 0.05. GO enrichment analysis revealed that these unique DEGs were significantly enriched in 90 GO terms, including 49 biological processes (BP), 16 cell compositions (CC), and 25 molecular functions (MF) (Figure 5C and Table S2). The main BP categories included the response to toxic substances (GO: 0009636), generation of precursor metabolites and energy (GO: 0006091), and response to wounding (GO: 0009611) during the early pathogen infection stage. Among the MF categories, the antioxidant activity (GO: 0016209) pathway was enriched with the largest number of genes. In the CC category, plastoglobules (GO: 0010287) encompassed the highest number of genes.

We performed a KEGG pathway analysis to determine the metabolic pathways activated by the pathogen infection. The 2,206 unique DEGs in the comparison of R0D_vs._R3D were significantly enriched in 29 biological pathways (*p* ≤ 0.05, Table S3), of which, 5 main pathways included “plant hormone signal transduction (ko04075),” “plant-pathogen interaction (ko04626),” “phenylpropanoid biosynthesis (ko00940),” “amino sugar and nucleotide sugar metabolism (ko00520),” and “MAPK signaling pathway–plant (ko04016)” (Figure 5D). We discovered that the significantly enriched pathways were associated with plant resistance, such as “plant-pathogen interaction (ko04626)” and “MAPK signaling pathway–plant (ko04016).” Taken together, these findings suggested that several fundamental resistance response-related pathways were activated in the resistance radish “GLX” after pathogen infection.

### Marker-Assisted Selection of CR Cultivar in Radish

**Figure 6:**
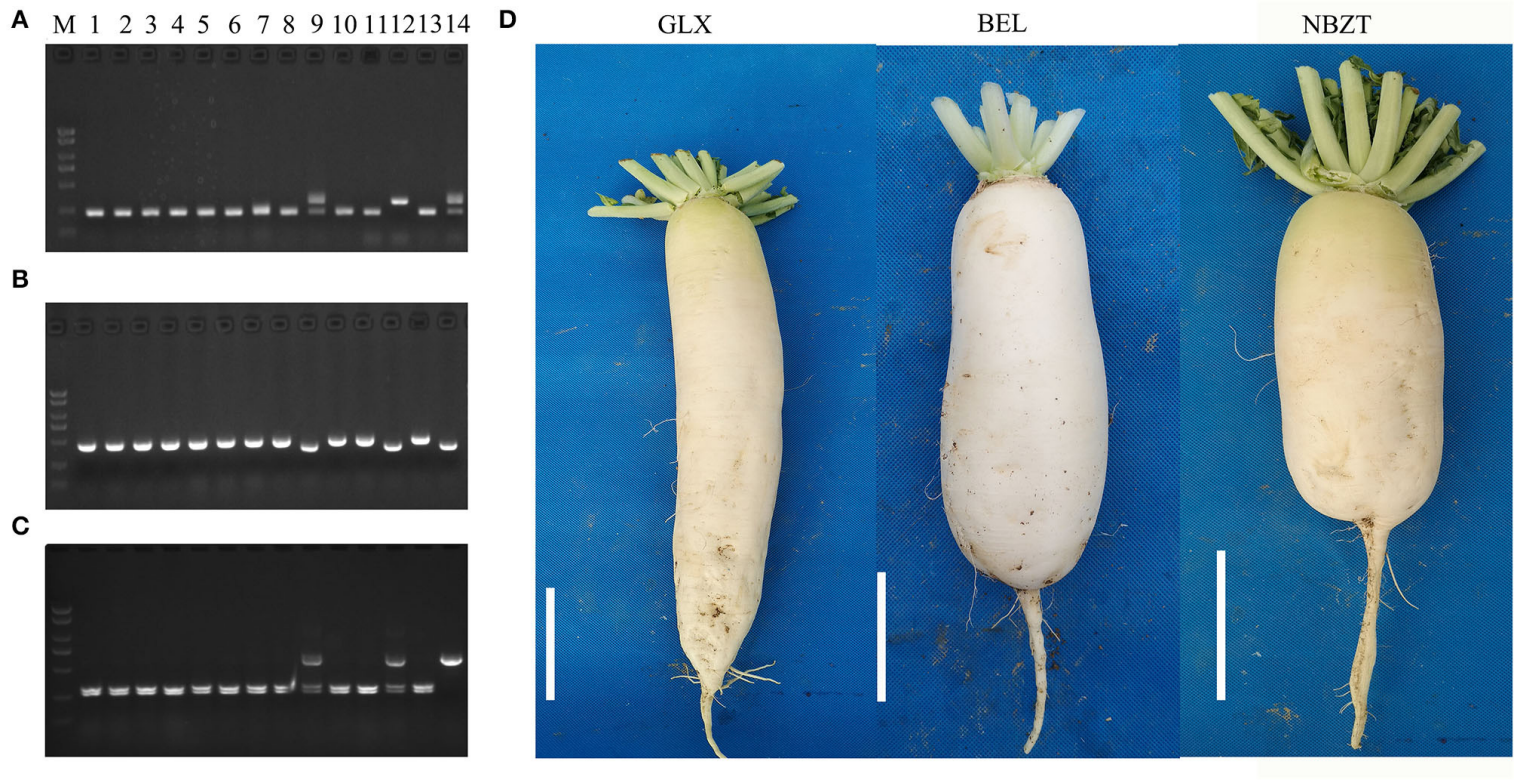
Development of molecular markers based on three major QTLs of clubroot resistance loci. **(A)** Development of InDel molecular marker HB167 in resistance locus Rs5. **(B)** Development of InDel molecular marker HB220 in resistance locus Rs9. **(C)** Marker-assisted selection of molecular marker HB321 in resistance locus *RsCr6* on chromosome Rs8. **(D)** Photograph of radish materials containing the resistance bands in three clubroot-resistant (CR) QTLs. The phenotypes of 14 radish breeding materials are presented in Table S4, with “S,” “R,” and “H” indicating susceptible, resistant, and heterozygous individuals, respectively. “M” represents the 2 kb marker. The numbers represent 14 radish breeding materials. “GLX,” “BEL,” and “NBZT” are the common name of three selected resistant clubroot materials. Bar scale = 5 cm.

Based on previous studies on radish clubroot resistance, three QTLs have been identified, including the novel locus identified in this study. Based on two previously reported QTLs, *Crs1* on chromosome Rs5 (Kamei et al., 2010) and *RsCr2* on chromosome Rs9 (Gan et al., 2019), we developed two insertion/deletion (InDel) markers, HB167 and HB220, closely related to *Crs1* and *RsCr2*, respectively. For *RsCr6*, we used the CAPS marker HB321 to assist molecular detection. Among 14 representative breeding materials (Figures 6A–C), “GLX,” “BEL,” and “NBZT” contained the resistance band in the close linkage markers. Thus, we considered these three accessions to be resistant. To validate this hypothesis, we examined the phenotypes of resistant radishes in a pathogen-containing field (Changyang, China) in the sprint of 2019–2021. Three breeding accessions exhibited a high clubroot resistance (Figure 6D, Table S4).

## Discussion

### New CR Locus in Radish

Huoshaoping, one of the main radish-producing areas, is seriously affected by clubroot disease (Gan et al., 2016). The prevalent pathotype in Huoshaoping has been identified as Pb10, according to the sinitic clubroot differential set (SCD) system (Pang et al., 2020). In this study, we identified a new locus named *RsCr6*, which was considered the main effect of the locus resistance to clubroot in radish. The resistant and susceptible parents (“GLX” and “XNQ,” respectively) and F_1_ were inoculated with the Pb10 strain, respectively. The results indicated that F_1_ plants exhibited no symptoms (Figure 1). Genetic analysis of resistant and susceptible parents (Figure 1), F_1_ plants, and F_2_ populations after inoculation showed that clubroot resistance was controlled by a dominant locus (Table 1). In this study, we used a BSA-seq approach, an effective method for rapidly obtaining linked target genes, Fu et al., 2019) based on whole-genome second-generation sequencing to map the resistance genes. The 823 progenies were expanded to ensure the accuracy of the results. Among these plants, disease-resistant individuals were planted in the field for investigation 6 weeks post-inoculation to ensure further resistance. If plants still showed prominent susceptible root clubs in the later period, they were counted as sensitive. In total, 50 samples from the R-pool were selected from 637 individual resistant plants (Table 1), planted in the field after further investigation, and redetermined to be resistant after inoculation. The S-pool comprised 50 seedlings with the highest level of grade 4 symptoms. The results showed that a single peak was identified on chromosome Rs8, and the candidate region was named *RsCr6* with a statistical significance at the level of *p*<*0.05* (Figure 2A). This is consistent with the results of the genetic analysis. In previous studies, two clubroot resistance QTLs, *Crs1*, and *RsCr2*, were identified on chromosomes Rs5 and Rs9, respectively (Kamei et al., 2010; Gan et al., 2019). The results suggested that a large part of the CR of radish is controlled by *Crs1*, and no other region showed logarithm of the odds (LOD) scores higher than the threshold in both tests (Kamei et al., 2010). *RsCr2* was repeatedly identified as the resistant locus on chromosome Rs9 (Gan et al., 2019). These findings indicate that the CR of radish may be controlled by multiple major QTLs. In this study, we detected a single peak as a candidate region anchored on chromosome Rs8 and designated as *RsCr6*, considered to be associated with clubroot resistance. This result differs from our previous report that resistance to clubroot is controlled by multiple QTLs in radish (Gan et al., 2019). This difference may be attributed to the use of different resistant parents (“BJJ” vs. “GLX”) and different pathotypes [race4 (Williams, 1996) vs. pathotype pb10 (Pang et al., 2020)].

### Molecular Marker Development for Breeding CR Cultivar

An economical approach to control clubroot disease is utilizing resistant materials and breeding resistant cultivars (Pang et al., 2018). With the development of molecular marker technology, MAS is an effective tool for screening for disease resistance (Collins et al., 2018). To date, a series of molecular markers have been developed and applied to select the new resistant germplasm resources (Kamei et al., 2010; Ueno et al., 2012; Wang et al., 2012; Hatakeyama et al., 2013; Zhang et al., 2014). To take advantage of multiple resistance resources, we tried to pyramid three resistance QTLs to breed a new cultivar in radish. Thus, we designed and developed three closely linked molecular markers, HB167, HB220, and HB321, for QTL located in Rs5, Rs9, and Rs8, respectively. The genotype of 14 radish accessions results revealed that three accessions, “GLX,” “BEL,” and “NBZT” contained resistant binds in three markers (Figure 6). As expected, the phenotypes of the 14 radish accessions were consistent with the genotyping results, which were confirmed over 5 years in the famous clubroot epidemic area of Huoshaoping in China (Changyang, Hubei Province). Interestingly, we discovered a radish cultivar, namely, “SQY” showing clubroot resistance in the greenhouse and field, but no clubroot resistance was identified in three QTLs (Table S4). Therefore, we hypothesized that a novel gene and/or more genes might confer clubroot resistance in radish, further confirmed in a recent study by Wang J. L. et al. (2022). The clubroot resistance gene *CRa* in *B. rapa* was aligned to a homologous region on chromosome Rs4 in radish, which has not been previously reported. During the investigation of radish clubroot, MAS, in combination with traditional cross-breeding, was employed to breed CR cultivars in radish. In the last 5 years, two CR cultivars, nominated as “Chuyu No.1” and “Chuyu No.2,” were developed in our laboratory (Cui et al., 2020). To our knowledge, two radish cultivars have been applied in several critical clubroot epidemic areas of China, such as Changyang and Wulong in Chongqing Province.

### Candidate Gene *RsCr6* and Resistance Response Pathways Activated by Clubroot Infection

Transcriptome analysis of Brassica species at different time points after inoculation with *P. brassicae* has been previously reported (Fu et al., 2019; Wang Q. B. et al., 2022). In this study, 2,206 unique DEGs were detected in R0D_vs._R3D (Figure 4D), based on the difference in genetic background between the parents. These results indicate that these genes might be involved in the response to clubroot resistance in radish. The top five biological process subcategories, which contained cellular processes, metabolic processes, single-organism processes, responses to stimuli, and biological regulation, were consistent with previous research reports (Fu et al., 2019). According to the gene expression in the region of *RsCr6, R120263140*, which encodes a MIZU-KUSSEI-like protein, was downregulated at 3 dpi in the resistant parent GLX (Table 2), which is considered an essential gene responsible for clubroot resistance in radish. Furthermore, *R120263070* encoded the protein RPP1, which has a high likelihood of inducing disease resistance. It was thus considered a strong candidate gene related to *RsCr6*. Although no differential expressions were observed in the two pairwise comparisons, it was chosen to avoid unpredictable factors. However, *R120263160* was not a candidate gene because, although it showed a similar expression trend to *R120263140*, the difference in expression level was not significant. These results collectively indicated that the candidate genes, *R120263140* and *R120263070*, might play a key role in the stage of *P. brassicae* infection, a hypothesis that will be tested in future work. Our study provides a solid foundation for investigating molecular mechanisms underlying clubroot resistance in radish and sheds light on radish breeding programs in the near future.

## Data Availability Statement

The datasets presented in this study can be found in online repositories. The names of the repository/repositories and accession number(s) can be found at: NCCB NGDC Genome Sequence Archive, accession numbers: PRJCA008464 and PRJCA008465.

## Author Contributions

CG and XD conceived and managed the project. CG investigated the genetics of radish and managed the plant materials. CY performed the data analysis. WP, LC, PF, XY, ZQ, and MZ analyzed the results. CG wrote the manuscript with the help from XD, WP, ZP, and CY. All authors reviewed and approved this manuscript.

## Funding

This study was supported by the National Key Research and Development Program of China (2021YFD1600300), the China Agriculture Research System of MOF and MARA (CARS-23-B-06), the Key Research and Development Program of Hubei Province, China (grant numbers 2020BBA037 and 2020BBB083), and the Liaoning Natural Science Foundation (2021-MS-229).

## Conflict of Interest

The authors declare that the research was conducted in the absence of any commercial or financial relationships that could be construed as a potential conflict of interest. The handling Editor CZ is currently organizing a research topic with the author ZP.

## Publisher's Note

All claims expressed in this article are solely those of the authors and do not necessarily represent those of their affiliated organizations, or those of the publisher, the editors and the reviewers. Any product that may be evaluated in this article, or claim that may be made by its manufacturer, is not guaranteed or endorsed by the publisher.
