# Supplementary material for: Identification of Novel Locus RsCr6 Related to Clubroot Resistance in Radish (Raphanus sativus L.)
Source: Front Plant Sci. 2022 May 19;13:866211. doi: 10.3389/fpls.2022.866211 (PMC9161170; doi:10.3389/fpls.2022.866211)
Supplement: Supplementary file 1 [file Table_1.DOCX]

**Table S1 The information of sequencing two pools**

| **Sample** | **Clean Reads** | **Clean Bases** | **GC(%)** | **Q20(%)** | **Overall Alignment Rate (%)** |
| --- | --- | --- | --- | --- | --- |
| R-pool | 62,888,180 | 18,295,677,463 | 37.72 | 96.20 | 87.50 |
| S-pool | 60,251,049 | 17,582,276,637 | 38.10 | 96.45 | 85.88 |
