# Supplementary material for: Identification of Novel Locus RsCr6 Related to Clubroot Resistance in Radish (Raphanus sativus L.)
Source: Front Plant Sci. 2022 May 19;13:866211. doi: 10.3389/fpls.2022.866211 (PMC9161170; doi:10.3389/fpls.2022.866211)
Supplement: Supplementary file 4 [file Table_4.DOCX]

**Table S4 The information of genotypes and corresponding phenotypes of fourteen radish accessions in three clubroot QTLs**

| ID | Name | Phenotype in field | HB167 | HB220 | HB321 |
| --- | --- | --- | --- | --- | --- |
|  |  |  |  |  |  |
| 1 | SQY | R | S | S | S |
| 2 | WXQ | S | S | S | S |
| 3 | SLB | S | S | S | S |
| 4 | WWB | S | S | S | S |
| 5 | HB | S | S | S | S |
| 6 | QZ-16 | S | S | S | S |
| 7 | LYLB | S | S | S | S |
| 8 | BQ | S | S | S | S |
| 9 | GLX | R | H | R | H |
| 10 | CYH | S | S | S | S |
| 11 | L47 | S | S | S | S |
| 12 | BEL | R | R | R | H |
| 13 | QZ-16-3 | S | S | S | S |
| 14 | NBZT | R | H | R | R |
