# Supplementary material for: Identification of Novel Locus RsCr6 Related to Clubroot Resistance in Radish (Raphanus sativus L.)
Source: Front Plant Sci. 2022 May 19;13:866211. doi: 10.3389/fpls.2022.866211 (PMC9161170; doi:10.3389/fpls.2022.866211)
Supplement: Supplementary file 5 [file Table_5.DOCX]

**Table S5 Molecular markers for mapping of *RsCr6* in Rs8**

| **Name** | **Primers** | **Positions (Rs8, bp)** | **Types of molecular markers** |
| --- | --- | --- | --- |
| HABS289-F | GTAAGCAAGAAGGACGGGTA | 668602 | CAPS(HindIII) |
| HABS289-R | ACATTAGTTGTGTTTTCGCCT |  |  |
| HABS290-F | GCAAACCAGTGAAGGATGAC | 2537942 | CAPS(HindIII) |
| HABS290-R | GTCCGGTTCTTGTTCCAGTA |  |  |
| HABS285-F | TGGAGGTTTCATCTTGCCTT | 8516279 | CAPS (EcoRI) |
| HABS285-R | TCAATGTCTAGTCAGTCGACC |  |  |
| HABS294-F | GTCAAGCTGAAACGCAATCT | 8992453 | CAPS(HindIII) |
| HABS294-R | ATGTATGATGTGGACAAGTG |  |  |
| HABS299-F | CGGATATCCATTTGGCTTTGG | 9652051 | CAPS (BamHI) |
| HABS299-R | CTGTCCGTTTTATTAAAAGTGT |  |  |
| HABS291-F | AAGAAGCGTCTTCAGGAAGT | 5484870 | CAPS (HindIII) |
| HABS291-R | TTGCTGTAGGAACGGTTGAA |  |  |
| HABS312-F | GGTCATCCGCATATTGTTGA | 5600813 | CAPS (HinfI) |
| HABS312-R | TTAGTCTGCACACACACAAG |  |  |
| HABS314-F | AATACTCTCAGTTTGGCAAC | 5892879 | CAPS (HpaII) |
| HABS314-R | CTCTCCATCCAGATGAGTGT |  |  |
| HABS318-F | GCTAAGCTCTGGTGTAGTGA | 6294498 | CAPS (XbaI) |
| HABS318-R | TGAGGAAAGAGCCTGTCTTC |  |  |
| HABS319-F | ACATGGAGTCGGTGAAGATC | 6378664 | CAPS (HindIII) |
| HABS319-R | TGGGCCCAAAACAAGCTATA |  |  |
| HABS321-F | ACAAAGGAACAATTCAACGA | 6681758 | CAPS (TaqI) |
| HABS321-R | ACTTCAGGCAAGTCAAGGAA |  |  |
| HABS331-F | TGAAAGTGAAGCACGTTAGC | 6773184 | CAPS (NdeI) |
| HABS331-R | CCTTTTCCATCGGTTTAGGC |  |  |
| HABS334-F | TCTCCGATAAATCCGACGAC | 6947176 | CAPS (DraI) |
| HABS334-R | AGTTACAAAAGCAAGTCGAAGT |  |  |
| HABS324-F | AGAATGTGGATTACTTTGCTGA | 6958261 | CAPS (HinfI) |
| HABS324-R | ACGGAGGGAGTATAATAAACT |  |  |
| HABS327-F | CCTTCGACTCCATCATACCC | 7127221 | CAPS (BsuRI) |
| HABS327-R | AGAAAGAGAATATTCGGACACA |  |  |
| HABS293-F | TTTTCAAAGAAACAGAGCAA | 7307784 | CAPS (HindIII) |
| HABS293-R | GTGAGAAAGATGTATGGCACC |  |  |
| HABS284-F | AGCTACCAAAGAATGCCTGA | 7745409 | CAPS (EcoRI) |
| HABS284-R | GGCTGGTTGTTACAGGAAAG |  |  |
